# Supplementary material for: A Humanized Yeast Phenomic Model of Deoxycytidine Kinase to Predict Genetic Buffering of Nucleoside Analog Cytotoxicity
Source: Genes (Basel). 2019 Sep 30;10(10):770. doi: 10.3390/genes10100770 (PMC6826991; doi:10.3390/genes10100770)
Supplement: Supplementary file 1 [file genes-10-00770-s001.zip › Additional_Files/Additional_File7_GOTermHeatmaps.zip!/Additional_File7_GOTermHeatmaps/DNA_replication_origin_binding.pdf]

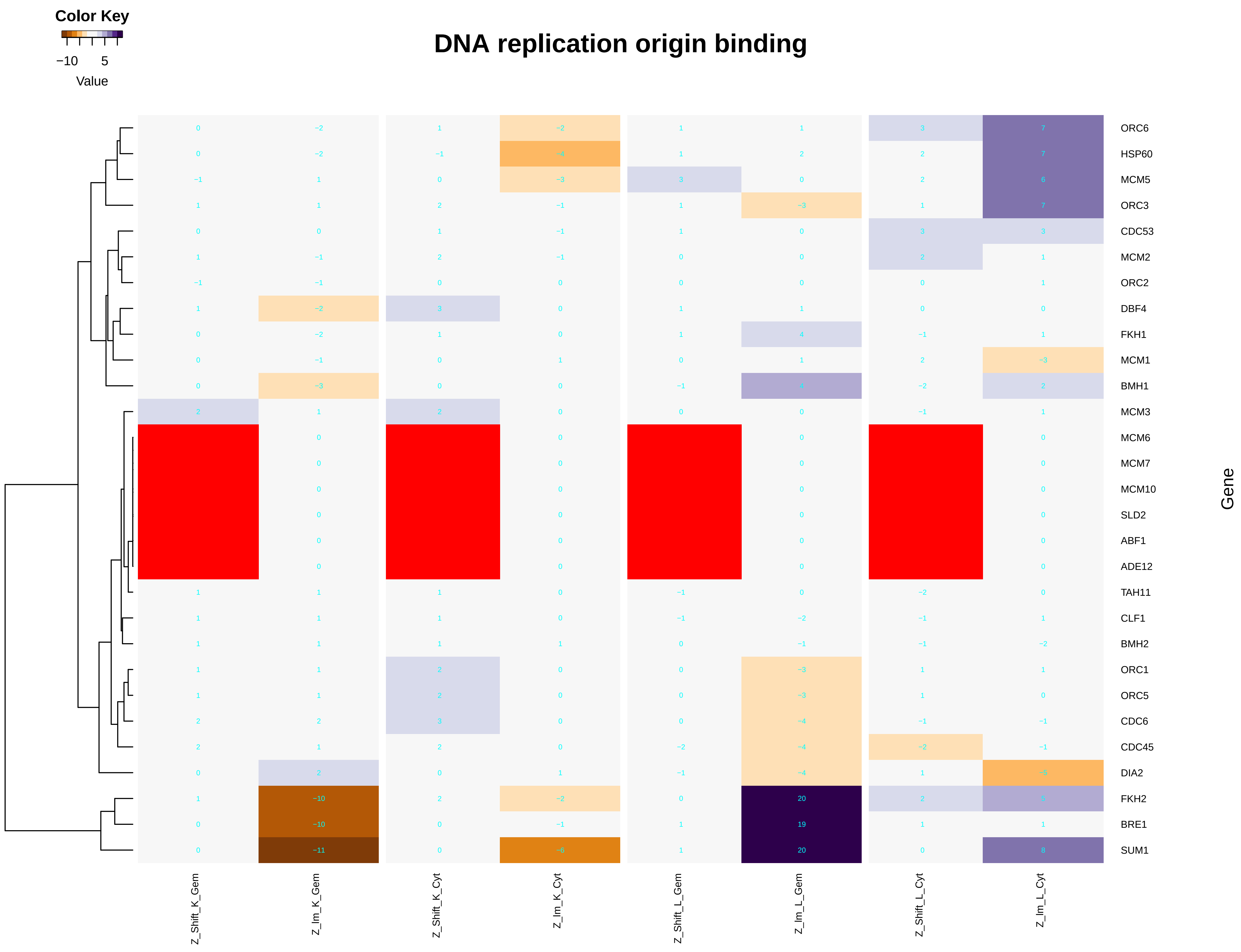
Color Key
−10
5
Value
DNA replication origin binding
ORC6
HSP60
MCM5
ORC3
CDC53
MCM2
ORC2
DBF4
FKH1
MCM1
BMH1
MCM3
MCM6
MCM7
MCM10
SLD2
ABF1
ADE12
TAH11
CLF1
BMH2
ORC1
ORC5
CDC6
CDC45
DIA2
FKH2
BRE1
SUM1
Gene
Z_Shift_K_Gem
Z_Im_K_Gem
Z_Shift_K_Cyt
Z_Im_K_Cyt
Z_Shift_L_Gem
Z_Im_L_Gem
Z_Shift_L_Cyt
Z_Im_L_Cyt
